# Polypoid Gallbladder Lesion in the Context of Renal Cell Carcinoma: Is Laparoscopic Cholecystectomy a Reasonable Option?

**DOI:** 10.1177/2324709613499008

**Published:** 2013-07-23

**Authors:** Barbara Seeliger, Cosimo Callari, Michele Diana, Didier Mutter, Jacques Marescaux

**Affiliations:** 1 University Hospital of Strasbourg, Strasbourg, France

**Keywords:** gallbladder metastasis, renal cell carcinoma, laparoscopic cholecystectomy

## Abstract

*Introduction*. The only curative therapeutic approach for renal cell carcinoma (RCC) is surgery. Laparoscopic surgery for RCC has become an established surgical procedure with equivalent cancer-free survival rate, following the same surgical oncological principles as open surgery. Metastatic RCC of the gallbladder is a rare phenomenon. Hence, there are few reports regarding their management. *Case Presentation*. We report 2 cases of gallbladder metastasis from clear cell RCC treated by laparoscopic cholecystectomy. The first case was that of a 44-year-old male patient who underwent palliative cholecystectomy, the second case was that of an 83-year-old female patient who is doing well 55 months after surgery without evidence of disease recurrence. *Conclusion*. The outcome allows us to demonstrate the interest of surgical resection of RCC metastases in the gallbladder by laparoscopic cholecystectomy, respecting surgical oncological principles. Laparoscopic resection of an uncommon gallbladder metastasis can provide long-term favorable outcome.

## Introduction

Renal cell carcinoma (RCC) represents the ninth most common malignancy in Europe, clear cell carcinoma being the main histological type (80% to 90%),^1^ The gallbladder is a rare site of distant metastases. At necropsies, Weiss et al^2^ found only 4 gallbladder metastases among 687 patients with RCC. Hence, there are few reports regarding their management.

We report 2 cases of gallbladder metastasis from clear cell RCC that were treated by laparoscopic cholecystectomy.

## Case 1

**Figure 1.**
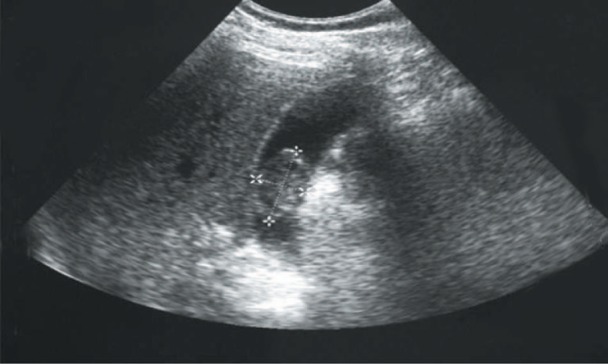
Case 1. Ultrasonography revealing a polypoid intraluminal echogenic nonshadowing gallbladder structure.

**Figure 2.**
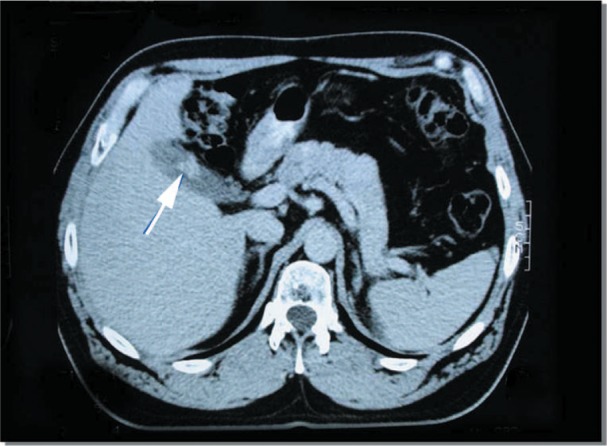
Case 1. Axial computed tomography image showing the polypoid structure adherent to the gallbladder wall (arrow).

**Figure 3.**
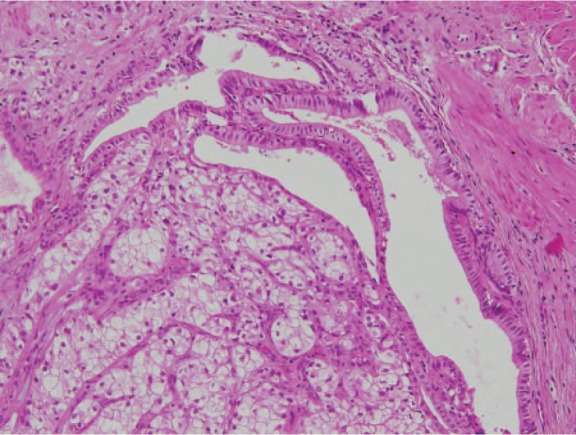
Case 1. Representative micrograph from the polypoid mass in the gallbladder shows metastatic clear cell renal cell carcinoma with overlaying gallbladder mucosa (hematoxylin–eosin stain, original magnification 20×). By courtesy of Alina Onea, MD, Department of Pathology, University Hospital of Strasbourg, Hôpital de Hautepierre, France.

A 44-year-old man underwent cytoreductive left radical nephrectomy for RCC with initial metastatic spread to mediastinum, lungs, bladder, and gallbladder. Primary surgery was followed by immunotherapy with interferon-α (IFN-α) and interleukin 2 (IL-2), resulting in a partial response of the pulmonary and mediastinal lesions and a complete response in the bladder. However, the gallbladder polypoid mass increased in size from the initial 1.5 cm to 3.0 cm diameter 12 months later on follow-up imaging (Figures 1 and 2), and the patient developed right upper quadrant pain. The patient subsequently underwent laparoscopic cholecystectomy. Histological analysis showed a clear cell carcinoma polypoid sessile metastasis sharing similar histological RCC features (Figure 3). The adjacent cystic lymph node was free of tumor invasion. The patient had an uneventful immediate postoperative course and was discharged on postoperative day 1.

Interferon treatment was restarted 3 months later, and the patient experienced approximately 6 months free of tumor progression. Further clinical course was marked by cerebral metastases onset requiring radiotherapy and additional treatment with Tamoxifen for multisystemic metastatic progression (additional hepatic metastases, peripheral lymphadenopathy, and disseminated cutaneous nodules). An atraumatic fracture of the right femoral neck was due to histologically confirmed osseous RCC spread. The patient died at age 48 due to deterioration of his general condition, 41 months after polymetastatic RCC diagnosis and 22 months after cholecystectomy.

## Case 2

**Figure 4.**
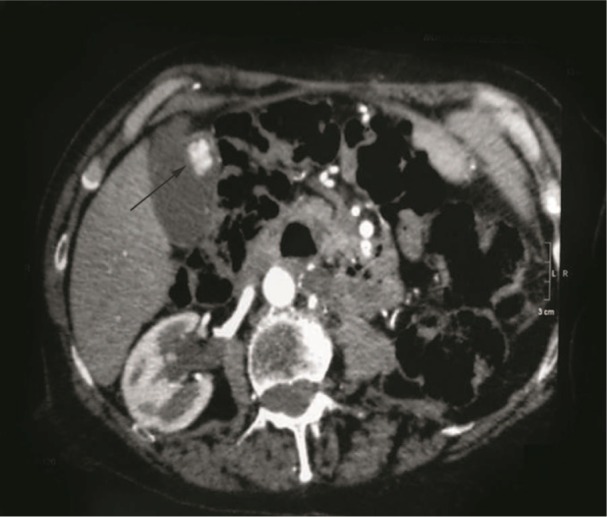
Case 2. Computed tomography image after intravenous contrast enhancement reveals an intraluminal hyperdense enhancing mass (arrow).

**Figure 5.**
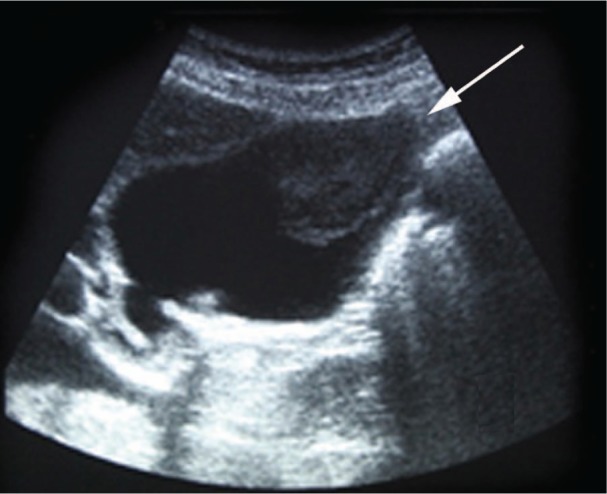
Case 2. Ultrasonography image with immobile mass in the fundus (arrow).

**Figure 6.**
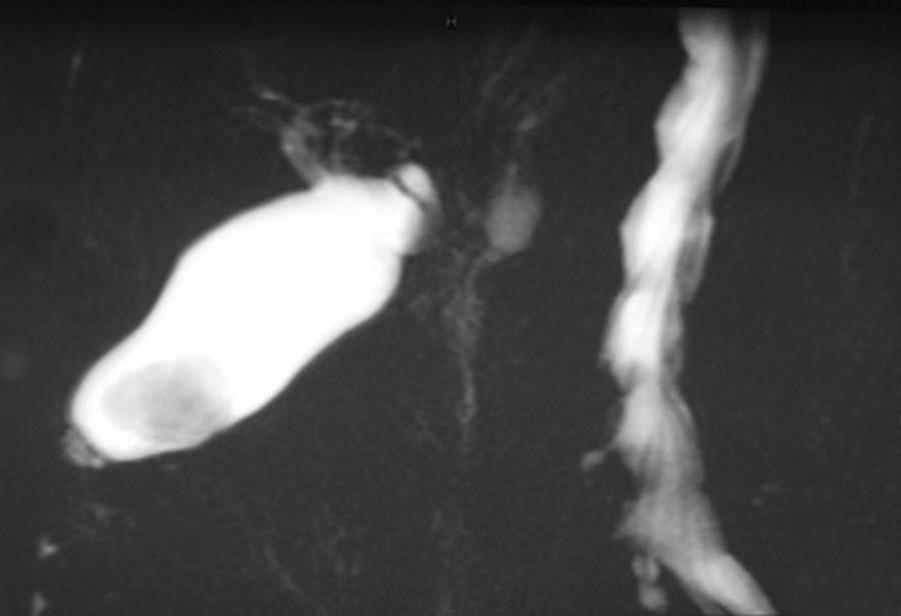
Case 2. Three-dimensional reconstruction of axial, T2-weighted magnetic resonance imaging revealing a solid structure (arrow) adhering to the inner gallbladder surface, surrounded by bile.

**Figure 7.**
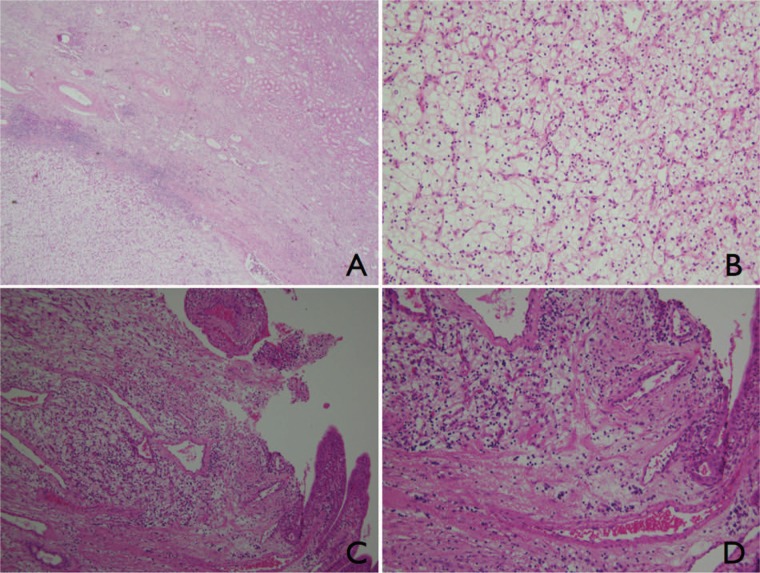
Case 2. Representative micrographs, hematoxylin–eosin stains. (A) Low-power view of the primary renal mass (4×). (B) Magnification (20×) of the renal mass showing clear cell RCC cells with clear, abundant cytoplasm. (C) Section from the gallbladder mass reveals a carcinoma with clear cell features (10×). (D) Magnification (20×) of the gallbladder mass, representing RCC metastasis with clear cell type cells and abundant vascularization in the stroma. By courtesy of Alina Onea, MD, Department of Pathology, University Hospital of Strasbourg, Hôpital de Hautepierre, France.

An 83-year-old woman underwent laparoscopic left radical nephrectomy for localized RCC. Twelve months later, a follow-up computed tomography scan of chest/abdomen/pelvis showed a solitary suspect 15-mm nodule in the gallbladder (Figure 4). It clearly increased in size 8 weeks later, when ultrasound scan (Figure 5) showed a polypoid lesion in the fundus measuring 30 mm, hypervascularized on Doppler sonography. There was no invasion of adjacent structures. Magnetic resonance imaging confirmed the polypoid mass at the gallbladder fundus stuck to the inner gallbladder surface with strong contrast enhancement (Figure 6). Ultrasound and magnetic resonance imaging visualized a concomitant solitary gallstone. The patient underwent laparoscopic cholecystectomy. Histopathological analysis showed a polypoid tumor of 2.2 × 2.0 cm in the gallbladder fundus representing RCC metastasis with clear cell type cells and abundant vascularization in the stroma (Figure 7). The adjacent cystic lymph node was free of tumor. The postoperative course was uneventful and the patient is recurrence free at 55-month follow-up without any additional therapy.

## Discussion

A clear cell metastatic deposit in the gallbladder occurs in a context of either synchronous primary renal tumor or metachronously in a past history of nephrectomy for RCC. Primitive clear cell carcinomas affecting the gallbladder are very uncommon neoplasms that can closely mimic metastatic deposits of RCC.^3^ Primary clear cell carcinomas of the gallbladder can demonstrate locally advanced growth,^3^ requiring a more extensive surgery than cholecystectomy.

In both our cases, the typical hypervascular polypoid gallbladder neoplasm in a context of RCC evoked a metastatic deposit. RCC gallbladder metastases are typically intraluminal polypoid masses, mainly confined to the mucosa or muscular layer of the gallbladder wall.^4^ Congruent with previous findings, our cases did not show external gallbladder surface involvement.

The only curative therapeutic approach for RCC is surgery, achieving excision of all tumor deposits. Laparoscopic surgery for RCC has become an established surgical procedure with equivalent cancer-free survival rate, following the same surgical oncological principles as open surgery.^1^

Laparoscopic cholecystectomy has evolved into a standard procedure in the management of benign disease. Cholecystectomy in RCC gallbladder metastases has been reported,^5,6^ but remains rare. The role and potential lack of adverse effects of laparoscopic management in the context of malignant disease has not been determined yet. In a precedent study, laparoscopic management did not lead to port-site seeding, and it even had a beneficial effect on local growth. Compared to laparoscopy, tumor manipulation was observed to lead to significantly increased tumor growth in the laparotomy group.^7^

The course of metastatic RCC is variable between rapid progression and long-lasting stabilization or spontaneous remission. Therefore, immune mechanisms are assumed to influence the clinical outcome.^8^ We experienced divergent responses to immunotherapy in different organs affected by RCC metastasis in our first patient. A lower therapeutic effect may be related to lesser tissue vascularization. Responses to IFN-α have been most frequently seen in pulmonary metastases and to a lesser degree in the lymph nodes.^8^ As cytokine therapy with IFN-α and IL-2 only nonspecifically activates the immune system, the mechanism of action is not well defined. Specific molecular targeted therapies are currently evolving and leading to advances in understanding and treatment of metastatic RCC.^9^

We report laparoscopic cholecystectomy for RCC metastasectomy including follow-up and outcome. In our first patient, palliative cholecystectomy was performed in the late 1990s for local tumor growth under nonspecific immunotherapy in the context of polymetastatic disease. In contrast, in our second patient the intention was curative. There was a single metastatic localization and the laparoscopic approach led to an excellent oncologic result with a disease-free survival of 55 months as of now, the patient being alive and fit. In both cases, no local recurrence related to the resection was observed.

Simple laparoscopic cholecystectomy may even be curative as shown by the disease-free clinical course of one of our patients following complete solitary gallbladder metastasis resection. Although limited, our experience supports the hypothesis^10^ that cholecystectomy may increase survival for patients presenting with solitary gallbladder metastasis and may serve as curative resection. Outcomes for patients with solitary gallbladder metastasis who underwent cholecystectomy appear similar to other solitary metastatic sites treated with metastasectomy.^11^

## Conclusion

RCC metastasis of the gallbladder is a rare phenomenon that can occur synchronously as well as metachronously. Cholecystectomy without extended resection seems to be sufficient for metastatic RCC deposits in the gallbladder as they occur without invasive tendency. In the extraordinarily rare case of an isolated gallbladder metastasis, our case demonstrates the benefit of minimally invasive curative cholecystectomy.

Consequently, laparoscopic cholecystectomy should be the preferred option in the treatment of RCC metastasis provided that general surgical oncologic principles are respected.
